# Generation gap, learning from the experience of compulsory remote architectural design studio

**DOI:** 10.1186/s41239-022-00345-7

**Published:** 2022-08-03

**Authors:** Aminreza Iranmanesh, Zeynep Onur

**Affiliations:** 1 Faculty of Architecture and Fine Arts, Final International University, Girne, TRNC Turkey; 2 Faculty of Architecture, Near East University, Nicosia, TRNC Turkey

**Keywords:** Virtual studio, Architecture, Pedagogy, Distance education, Generation gap, Design studio, COVID-19

## Abstract

Physical design studio has been the mainstream method of architectural pedagogy for more than a century. Although the past two decades have brought forth emerging possibilities via advancements in digital communication, Virtual Design Studio (VDS) remained an experimental novelty until 2020. The interruption of face-to-face educational activities saw architecture schools facing a rapid paradigm shift because their studio-centred pedagogy retains intrinsic spatial qualities that are often attributed as critical dimensions of the learning process. This article explores the transition to the virtual design studio in a department of architecture after distance education became mainstream due to the global pandemic. The paper provides a comparison between students’ and lecturers’ points of view regarding different aspects of the virtual design studio. This addresses a potential generational gap concerning digital communication in a case study. A survey was administered to a group of architecture students who travelled back home and continued their education online and to the teachers of design studios who instructed VDS after the pandemic outbreak. The findings show the significant influence of effective communication, access to proper resources, maintenance of peer connections, and group works on the positive outcomes of the architectural design studio.

## Introduction

The repercussions of the COVID-19 pandemic affected all higher education disciplines—some more than others. Disciplines with lab work and hands-on pedagogical approaches faced significant challenges. In these disciplines, distance learning required calibration of the curriculum and etiquette of programs that were not originally designed to accommodate virtual classrooms (Neuwirth et al., 2020). Moreover, the abrupt move to virtual space during the global pandemic created a transition period for which many students and lecturers were not prepared.

In schools of architecture, almost universally, design studio stands at the heart of the learning process (McClean, 2009; Salama & Wilkinson, 2007). At first glance, it might seem that the transition to virtual space could be done easily for architectural studios; after all, the drawing and modelling can be done—and have been done for a while—via digital software. However, the design studio presents opportunities such as peer learning that might be difficult to reproduce in a virtual environment (Fleischmann, 2019; Kvan, 2001).

The conventional form of design studio has been influenced by the seminal work of Schön (1983) and is based on the transfer of knowledge from the master practitioner to the novices during the continuous process of ‘reflection in action’. This ‘learning by doing’ process takes place in the studio space while students observe and interact with the teacher and with one another. For Schön and Wiggins (1992), the material medium (pen, paper, and models) through which the reflective interaction takes place is a critical aspect of the architectural design studio. In this light, gestures, body language, collaborative work on physical models, layered drawings, and the socio-cultural atmosphere of design studio, are among the qualities that need to be considered when shifting to virtual space (Senyapili & Karakaya, 2009). A conundrum presents itself here. While today, the students of architecture learn and become fluent in the ways of digital fabrication, computer-aided design, and digital interaction, the teachers might not possess the same skillset to be an effective part of virtual space (Brown, 2006; Doyle & Senske, 2016). Thus, students seem to be more flexible in adapting to virtual studios, whereas the teachers might not find these emerging approaches effective or suitable for the design studio (Bayhan & Karaca, 2020). In such scenarios, the student might find only verbal recommendations that are not necessarily objective and even might be misleading if the teacher does not understand or is unable to communicate with the virtually presented material (Brown, 2006). This can have hidden strengths and weaknesses. It might empower self-dependent students that feel comfortable having fewer interventions and allow them to experience new approaches; however, it might limit the potential of extroverted students who thrive on interactions or those who require more objective tutoring (Offir et al., 2007; Park, 2020).

It became evident early on that transferring all design studios into virtual space is a challenging task compared to the transfer of the theory-based courses (Ibrahim et al., 2021). In the context of this study, most academic staff had very little experience with conducting a virtual design studio prior to the pandemic. Although all faculties, departments, and staff were equipped to shift to online educational platforms, the majority had never dealt with this type of teaching. This sudden transition brought the education of the architect, which was already being questioned, into urgent discourse. Arising questions included: What is the nature of distance education regarding architectural studios? What are suitable tools and mediums? What should the learning outcomes be, and how do these compare with the physical design studio?

Moreover, the attitudes toward remote education and VDS seem to be different when comparing students and teachers (Nespoli et al., 2021). Generation Z students, who are in school now, are children of the information age; They know the language of social media as a native tongue (Bayhan & Karaca, 2020; Seemiller & Grace, 2016), and it is part of how they perceive and interact with the world (Green & Hannon, 2007). The experimental study of Chen and You (2010) indicated students’ willingness to adapt and their positive attitude toward internet-assisted design studios. The teachers, on the other hand, represent the status quo; they are a conservative force that might be more reluctant to change (Mercader & Gairín, 2020). This gap shows itself more prominently in self-learning and the utilisation of new digital tools in the studio workflow. Today, students might often have more extensive knowledge of digital tools than their teachers might. These differences, in turn, pose a problem for the bi-directional communication and collaboration that are fundamental to the very definition of design studio (Emam et al., 2019). The interactive processes that are intrinsic to design studio pedagogy require flexibility and versatility in the mediums of presentation (Schön & Wiggins, 1992). The advent of digital communication in all stages of the design process calls for exploring new theoretical frameworks that can potentially complement the traditional pedagogical logic of the design studio (Oxman, 2008). When over-the-table interaction using material mediums is no longer viable, the successful conduct of interactive virtual studio becomes a skill that educators need to learn.

The gap in literature seems to be related to the speed of adoption of digital tools and media into the workflow of architectural studios without having concrete pedagogical frameworks designed to support their incorporation. The educators of design studios today, for the most part, might come from a traditional system that was built on interactions with physical materials (Oxman, 2008). Accordingly, more studies are needed to explore the pedagogical frameworks that can accommodate virtual space. The flexibility to adopt new interactive mediums seems to be critical when imagining what might become mainstream in the forthcoming decades. In 2020, the global pandemic highlighted these shortcomings—exposing the gap between educators’ and students’ approaches to architectural design studio courses.

This article discusses the transition to VDS in a higher education institution during the global pandemic. The study compares students’ and lecturers’ points of view regarding different aspects of VDS. It aims to provide insights into the basic and irreconcilable inconsistencies in the overall structure of education, which, to date, has not been resolved. The paper is organised into three sections. First, the study briefly examines the existing literature of architectural pedagogy and VDS. Second, it explores different aspects of the design studio in a case study to provide a comparison between teachers’ and students’ experiences in transitioning to the virtual studio. The case study also examines self-evaluation of the design outcomes of the studio from the perspectives of students and faculty members. For this part, the survey tried to identify the critical differences between physical and virtual design studios. Third, the study discusses the strengths and weaknesses of VDS in fostering studio culture and its potential future applications.

### Architectural design studio and emerging virtuality

For some time, the effectiveness of some of the traditional education methods and philosophies has been questioned in the literature (Dewey, 1986). Rancière (1991) challenged the pedagogical norms of education in the nineties. In Rancière’s book, *The Ignorant Schoolmaster*, the questions related to what teaching means—who can be taught, who needs to be taught, and what an individual, a group, or ultimately society should learn—were among the first challenges to the notion of effective education. The experiences and statements of Robinson and Aronica (2016) and Sugata et al. (2005) evoked captious questions, signalling that something was going wrong in education. The pandemic conditions made it urgent to address these issues in architectural pedagogy, especially since its goal is to foster creativity and innovation.

The architectural design studio is the core of architectural pedagogy (McClean, 2009). Contemporary architectural pedagogy still follows a scheme similar to that established by Beaux-Arts and Bauhaus (Salama & Wilkinson, 2007). Although the mediums of communication and workflow have changed significantly over time, the approach toward the conventional design studio has remained stationary for decades without addressing the emerging educational contexts (Hettithanthri & Hansen, 2021). This seems counterintuitive for a practice that prides itself on adaptability, creativity, and dealing with emerging concepts (Smith, 2011). Grover et al. (2020) argued that deep learning requires moving beyond the design studio’s critique sessions and exploring parallel methods. It seems that the main challenge of architectural pedagogy is retaining the intrinsic qualities of a well-established traditional structure while trying to improve it for the future (Wilkin, 2005). Reforming architectural pedagogy has been a central discussion point for the past two decades, especially in light of advancements in digital technology (Salama, 2016). The advent of digital tools and communication mediums has changed the terrain of the design studio. Fjeld (2005) argued that the survival of the studio model depends on defining new frameworks that take into account these changes. These changes must enable students to cope with the emerging circumstances of professional practice (Nicol & Pilling, 2005). With the accelerated development of emerging tools and methods, it seems that training students to benefit from these nuances by conducting independent research and self-learning could be an ideal complement to the design studio pedagogy (Saghafi et al., 2012).

The first implementation of VDS dates back to 1993, and ever since, VDS has been part of the discussions regarding architectural pedagogy (Schnabel & Kvan, 2001, 2001). The screen-sharing and simultaneous 3-dimensional collaborations in the virtual space are as old as Web2.0 (Maher & Simmoff, 1999). Even among the earliest attempts, it has shown consistently to have the potential for implementing a successful design studio, especially in expanding teamwork and community development (Bucinell et al., 1997; Saghafi et al., 2012; Wojtowicz, 1995).

Going back to Rancière’s (1991, p. 130) argument, 'learning without a master explicator' means empowering students by encouraging self-learning processes. Reading into the pathology of the design studio, it often happens that students focus more on satisfying the teacher by trying to fit their design narrative into the teacher’s, or the jury’s, framework (Aderonmu et al., 2017). Attoe and Mugerauer (1991) described the *excellent teacher* as a caring person—a *parent figure* who does not act as an all-knowing character who cannot be challenged, but rather, as someone who interacts and participates in the design process by asking questions and instigating dialectical discussions. Proactive teachers can make a difference even when the environmental factors are hostile toward learning (Lapeniene & Dumciene, 2014). It has been argued that practising architecture is not an adequate qualification for teaching it; the pedagogical skills for teaching a design studio cannot necessarily be obtained via professional practice (Goldschmidt et al., 2010; Rhowbotham, 1995). One of the problems in this regard, as indicated by Nicol (2000), is that the knowledge of architectural design teaching remains tacit and bound to the continuously interactive processes that are confined to the studio space.

Furthermore, the very notion of competition, with peers or self, as a long-lasting tradition of the design studio is meant to showcase the extent of creative possibilities and to look at ideas that might be considered radical even though the most creative outcomes might not always emerge victorious (Ellsworth, 2005, p. 9). A design studio, in its idealistic essence, would be set up to do the same—elicit the impossible. The jury-based evaluation system of design studio, however, often encourages students to stay on the safe side and to work for the jury instead of trying extraordinary design ideas (Webster, 2007).

### Peer learning and architectural studio

The learning outcomes of an architectural design studio are not merely the design product. Outcomes consist of a complex set of social, cultural, and spatial experiences that are intrinsic qualities of the studio environment. Dutton (1987) called this a *hidden curriculum,* which is the sum of students’ experiences and interactions in the design studio. Architectural design, at its core, is a social process; it requires communication and people skills. Design studio is the platform through which these skills are developed and tested (Vowles, 2005). Learning in the design studio happens via two interwoven processes: the formal feedback (critique) with the teacher (s) and the informal dialect among peers (McClean & Hourigan, 2013). The informal social structure of the studio facilitates peer learning, which is one of its strongest assets; this informal process facilitates internalisation of the studio social structure while retaining one’s individual character as a designer (Gray, 2013). Despite the fact that peer learning is not a predefined intentional process embedded in the curriculum, it can be argued that it is the most memorable and influential aspect of the design studio (Koch et al., 2002).

For Schön (1987), design studio provided a platform in which the learner could exercise *learning by doing* while interacting with others and experiencing *reflection in action*. This experience is sometimes referred to as the studio culture, and it is associated with productivity and deep learning processes (Vowles et al., 2012). The teacher must be aware and supportive of this peer-learning process and its potentials (McClean & Hourigan, 2013). It can be argued that the medium of communication among the students of today is significantly different from 20 years ago. Mobile digital communication and social media have created a new continuous stream of virtual interactions superimposed on the space. Moreover, most students that are in the studio were born into this technology and do not know a world without it (Pektaş, 2015). They are native speakers of this hybrid semi-digital presence. It can be argued that they might be better at utilising the opportunities the digital world has to offer as opposed to lecturers who previously only knew the physical design studio and virtual education remains a temporary necessity for them.

Having said that, VDS can generate its own culture for all generations; this culture is an identity that is in the process of becoming (Al-Qawasmi, 2005). From the standpoint of possibility, the advancements in communication mediums are blurring the boundaries between virtual and physical; children of the internet era are now the students of the design studio. They have been exposed to these emerging communication mediums for as long as they can remember. They speak the language of the internet fluently, perhaps better than their instructors do. Hence, they have the potential to invent a new studio culture.

### Design studios during the COVID-19 pandemic

It became evident in the literature covering COVID-19 and education that transition to virtual learning is a complex and challenging task that might render both positive and negative outcomes (Ahmad, Erqou, et al., 2020; Ahmad, Sosa, et al., 2020; Iranmanesh & Onur, 2021; Powell & McGuigan, 2021). Ceylan, Şahin, Seçmen, Somer, and Süher (2021) showed that the most prominent improvement in design studios during the pandemic appeared in the utilisation and learning of digital tools. Iranmanesh and Onur (2021) indicated that the development of independent self-learning processes is the major highlight of VDS. The need for integrating digital tools into the flow of design studio pedagogy seems to be a necessity that requires supportive training and adjustments. A study by Alawamleh, Al-Twait Lana, and Al-Saht Gharam (2020) showed that the most significant consequence of the VDS is the way it affects communication and interaction. This does not merely affect student–teacher interactions but also the informal student–student interactions that are a significant part of the architectural pedagogy (McClean & Hourigan, 2013).

VDS can potentially have a wider reach for both audience and participation (L. Ahmad, Erqou, et al., 2020; Ahmad, Sosa, et al., 2020); this can bring more people into the studio workflow despite their proximal unavailability. The trials of K. Ahmad et al. (2020), Ahmad, Sosa, et al. (2020)) showed that simulation of design studio space via Virtual Reality (VR) can have a positive impact on providing some qualities that the physical spaces offer. Nevertheless, this remains limited to schools that can provide such equipment and to teachers who are well versed in modelling and coding virtual realities. The question of access to proper infrastructure and tools becomes more prominent in VDS. For instance, Peimani and Kamalipour (2021) argued that the degree of access to suitable hardware/software might create inequity among students.

Pries et al. (2020) asserted that separating students from each other is toxic for creativity. Furthermore, space, which is the core medium of architecture, can become lost in a virtual classroom, leaving very little room for casual peer interactions. The interactions among students create moments of learning-through-teaching in which students assume the role of the teaching figure by providing critiques on their peers’ projects. This is an effective pedagogical tool and can be utilised even in non-studio-based courses (Wagner & Gansemer-Topf, 2005). The concerns regarding communication and peer-learning processes are not new topics. McLuckie and Topping (2004) argued that the mere act of providing a platform is not sufficient; the pedagogical principles of peer learning must be facilitated and explored when conducting remote education. Iranmanesh and Onur (2021) showed that during the COVID-19 period, the decline in peer learning was the most significant upset of VDS (also see Fleischmann, 2020). Cabral, Freeman, Sachs, Schmidt, and Gamez (2020) called the practice of maintaining and reforming the concept of community in the sudden transition to VDS one of the most important learning opportunities for emerging pedagogical approaches.

### The case study

The study was conducted in the Faculty of Architecture in one of the universities located in Cyprus. As the magnitude of the COVID-19 impacts unfolded, all educational activities were suspended on March 11, 2020. It was initially perceived as a short-term, temporary precaution that would soon resolve; yet, the day of resolution did not come quickly. The educational activities were resumed via the university’s remote learning platform in the following week.

In this case, different studios experimented with mutual communication via many different media, such as Facebook groups, Google Classrooms, WhatsApp groups, and Skype. Soon thereafter, all classes were unified using Google Meet and integrated with the remote learning platform of the school. The application provided easy screen sharing and automated recording tools.

**Fig. 1:**
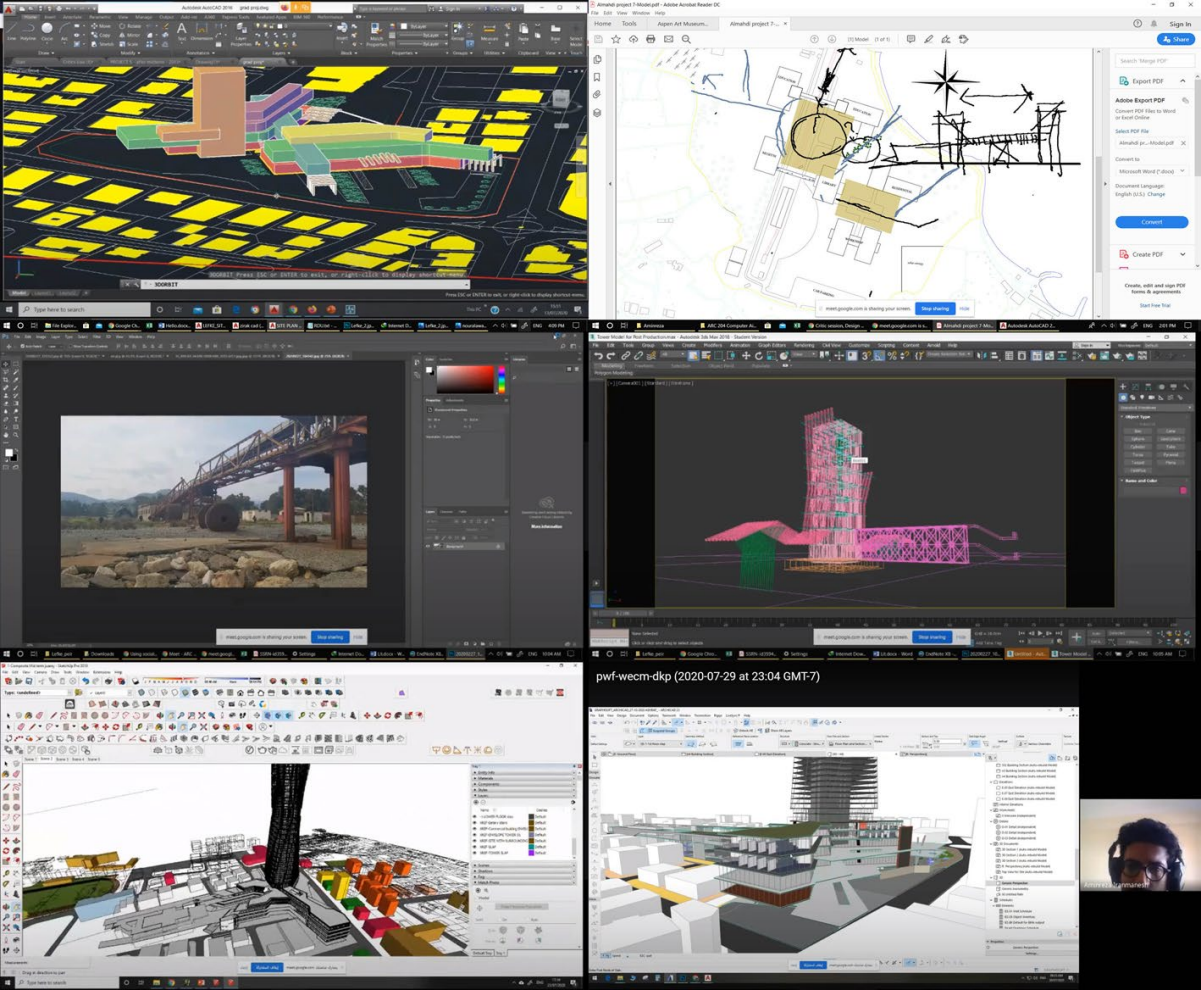
Variety of tools and methods reported in a single design studio

The screen sharing method was used as the main method of communication while conducting virtual classes in the school. To understand the difficulties of conducting a design studio via a screen, the effective methods of the physical studio must be revisited. From interactive work with physical models and drawing tools to the simplest tasks with body language like pointing at something, the physical space provides an effective and versatile setting for effective design tutorship. The virtual space, on the other hand, did not offer many of those flexibilities at first glance. At the same time, the students tended to use a variety of digital tools to present and showcase their design development in the virtual studio. For instance, in the graduation project critique sessions, the study found the usage of AutoCAD, Revit, 3DS Max, SketchUp, Rhinoceros 3D, ArchiCAD, Lumion, Twinmotion, Photoshop, and in one case 3D Blender (Fig. 1). Many of these tools were used before for design and display in the physical studio, but the critiques were typically given using gestures and supplementary drawing on paper. In this case, the screen sharing was one-directional, meaning that one contributor was always limited to verbal commands. This made it inevitable for the lecturer to also open the files and interact with the digital tools in order to provide feedback. One of the main problems with VDS presents itself here: many tutors might not be able to operate all of these tools.

**Fig. 2:**
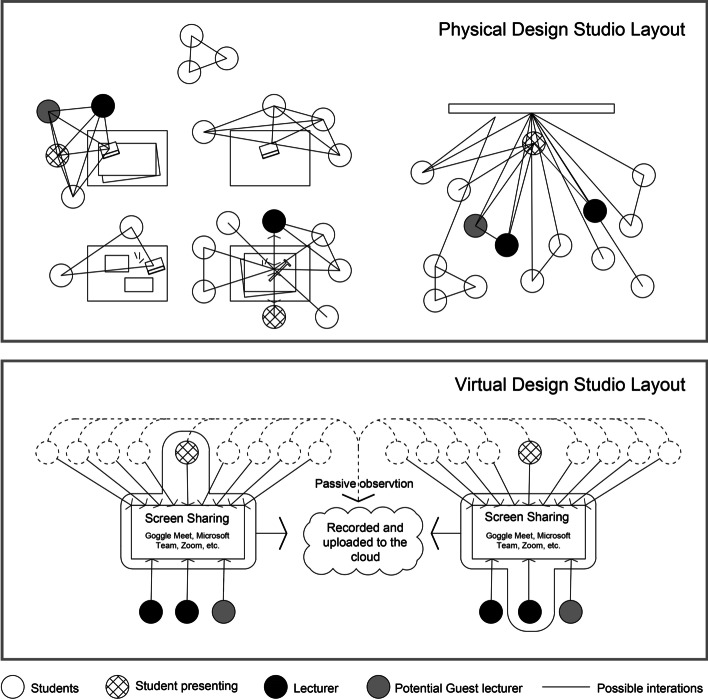
The general layout of the physical and virtual design studios in the case study

Another problem was the bi-directional nature of communication in the absence of a physical setting. The critique sessions, in this case, were being held mainly between the teacher and the student with others being mere observers (Fig. 2). The cacophony caused by multiple participants having their microphone on without being able to read the body language cues of others made it impractical to have engaging group discussions for the majority of each session. Communication seems to be among the critical variables in exploring the nature of VDS. Nevertheless, the virtual studio was not without its advantages, among which, the self-learning processes were observed in various aspects, such as research, digital communication, and time management. The availability of studio recording is another asset of virtual space. In this case, all recordings were made available to the students immediately after the class session concluded. The view count of these recording suggests that many students revisited their critique sessions. Accordingly, a systematic comparative study was conducted to explore the outcomes of VDS from the perspectives of students and lecturers.

### Data collection and filtration process

**Fig. 3:**
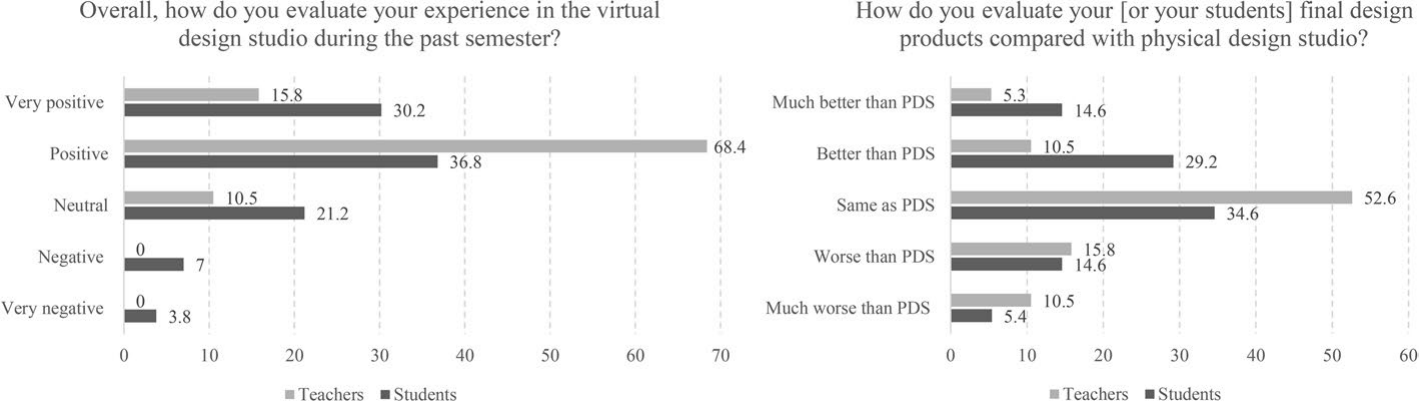
Left, overall evaluation of VDS experience; right, evaluation of final design outcomes. The reported numbers are actual percentages (rounded up) and include missing answers, if any

**Fig. 4:**
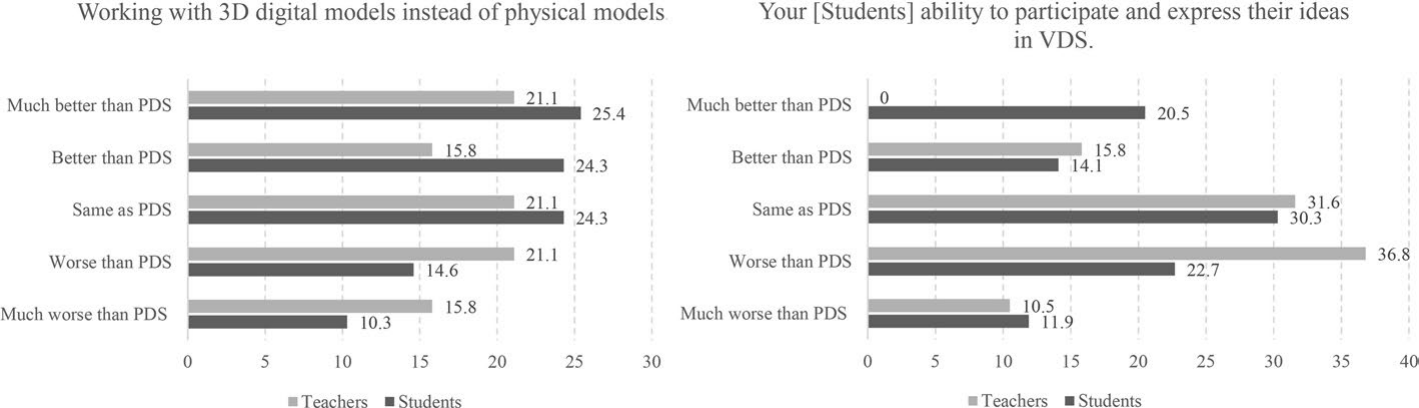
Left, the effectiveness of the 3D working model in VDS compared to PDS; right, students’ ability to express their ideas in VDS compared to PDS

**Fig. 5:**
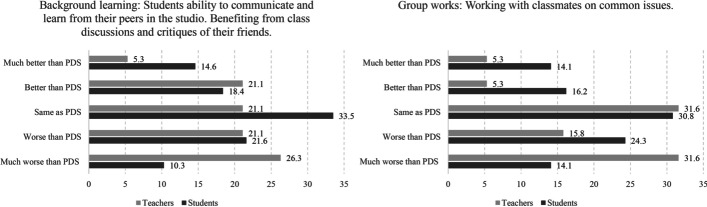
Left, background peer learning; right, conducting group work

**Fig. 6:**
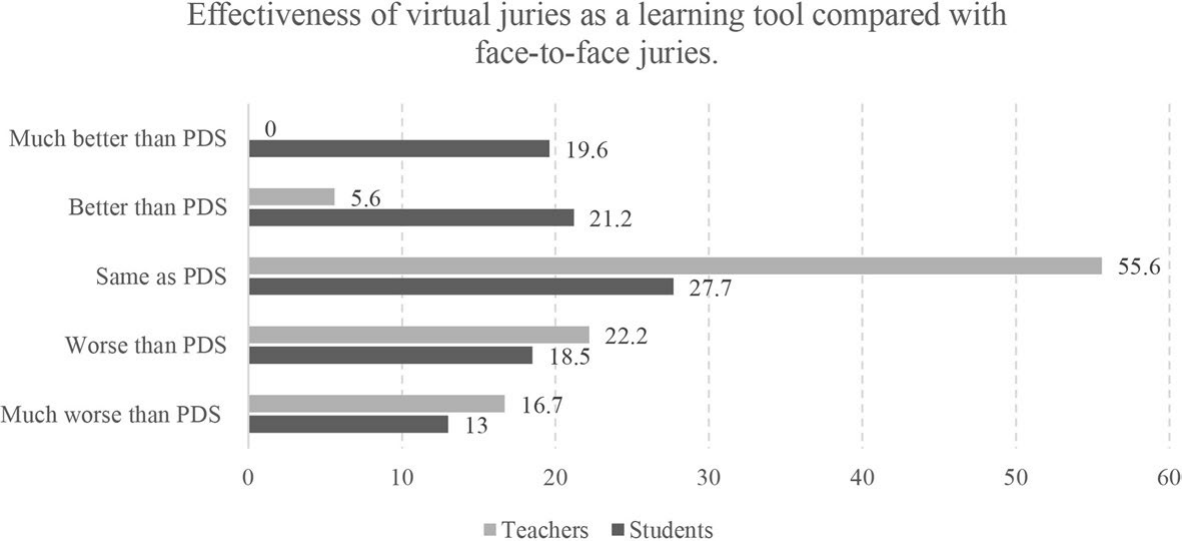
Effectiveness of virtual juries compared with physical juries

**Fig. 7:**
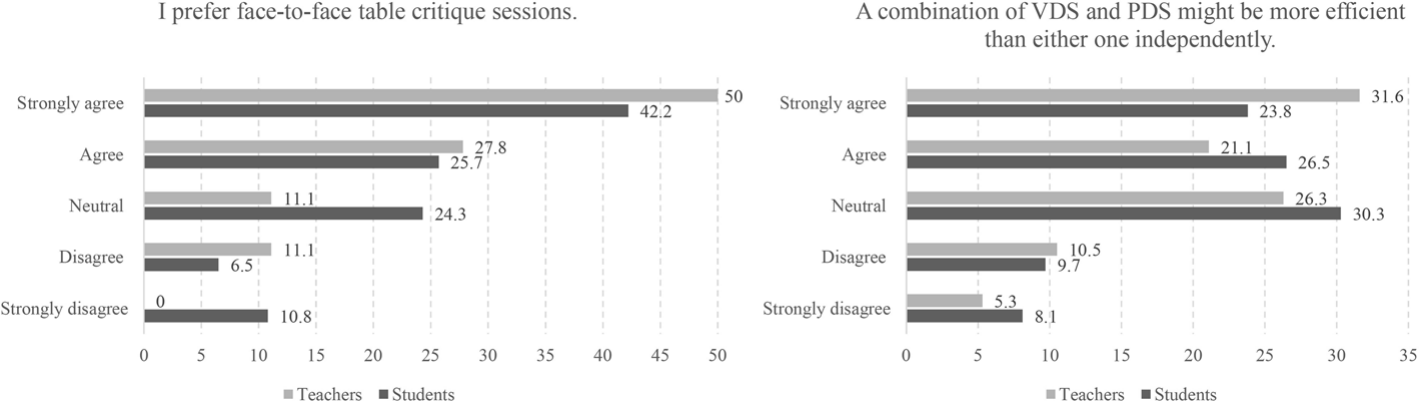
Left, the preference for face-to-face table critiques; right, the propensity for combining PDS and VDS

A survey was designed to address the parameters of the virtual studio in comparison to the physical studio. The survey consisted of 19 items in three sections:

- The survey explored the studio experience and self-evaluation of the design outcomes compared to the pre-pandemic physical studio (2 items: see Fig. 3).
- It addressed the main features of the design studio as it transitioned to virtual space. These items evaluate the efficiency of the critique sessions, teacher-student communications, peer learning, group activities, self-learning processes, integration of digital tools, and effectiveness of jury evaluation as a learning tool (15 items: see Figs. 4, 5 and 6 and Tables 1 and 2).
- It addressed the potential application of VDS in the future and the possibility of hybrid models from the perspectives of students and teachers (2 items: see Fig. 7).

**Table 1:** The Spearman rank correlation coefficient (ρ) between the overall experience of VDS and potential external factors

Spearman Rank Correlations Coefficient (ρ) (student responses)	Ordinal variables (5-point Likert scale): (1) Strongly disagree; (2) Disagree; (3) Neither agree nor disagree; (4) Agree; (5) Strongly agree		
	Internet connection speed was good enough for the virtual experience	I had access to a good computer to show my full potential	I had access to CAD software
Overall, how do you evaluate your experience in the online design studio in the past semester?			
ρ	0.322**	0.330**	0.479**
Sig. (2-tailed)	0.000	0.000	0.000
N	184	184	182
How do you evaluate your final design product when compared with the physical design studio?			
ρ	0.181*	0.337**	0.251**
Sig. (2-tailed)	0.015	0.000	0.001
N	181	181	179

**Correlation is significant at the 0.01 level (2-tailed)

*Correlation is significant at the 0.05 level (2-tailed)

**Table 2:** The effectiveness of digital presentation tools in the studio

	**Students**			**Teachers**		
	N	Mean	Coefficient of Variation (CV)	N	Mean	Coefficient of Variation (CV)
Scan of hand drawings and physical models	125	3.02	42.0%	11	3.27	38.9%
AutoCAD	152	4.41	22.2%	14	4.36	21.3%
SketchUp	118	3.69	36.4%	11	4.09	27.7%
Revit	66	3.17	41.9%	8	2.88	50.6%
ArchiCAD	87	3.71	36.7%	10	3.5	43.1%
3DS Max	82	3.6	35.9%	9	4.02	24.1%
Photoshop	125	3.98	25.9%	12	4.17	20.0%

The survey passed through the monitoring and approval process with the ethics committee of the host institution. The survey items are annotated in the following figures and headings as they appeared on the survey.

The Faculty of Architecture, in this case, offers three programs (architecture, interior architecture, and landscape design) in two languages (Turkish and English). At the end of the academic year of 2019–20 (summer), 715 active students were enrolled in design studios. These students consisted of 494 males (69%) and 221 females (31%) with the majority of them in the age range of 19 to 24 (mean = 20.73, Std. Deviation = 2.54). The Faculty of Architecture (English) consists of a majority of international and non-local students. In order to keep the contexts of the respondents uniform for this study, those who travelled back home and continued their education via the distance learning platform were selected as the target group (n = 367). This decision was motivated by the fact that many of those who stayed on the island were in contact with each other; therefore, their responses to the questions regarding peer learning might not have been purely representative of VDS. Students in their first year of the program were not included in the sample since they had had very limited prior experience with physical design studios (PDS). The sample was kept uniform considering students’ active semester. Accordingly, 185 active students voluntarily participated in the study after the semester was over. This number accounts for 25.8% of the total number of students and 51% of all students who travelled back to their home countries to continue with remote education (the sample meets the 95% confidence level). All of these students had a design studio course in the spring of 2020. In addition, 18 full-time staff members who were instructing design studio courses in the same period participated in the study (7 male and 11 female lecturers between the ages of 34 and 63, mean = 47.0, Std. Deviation = 8.89). The scale of measurement is a Likert scale of five. Most of the responses are comparative from 1 being ‘Much worse than PDS’ to 5 being ‘Much better than PDS’.

## Results and analysis

The majority in both groups (students and teachers) evaluated VDS as a positive experience (Fig. 3). This, however, might have been influenced by the amount of free time gained by not having to commute to work/education and the overall lower cost of VDS compared to PDS. In fact, this is the only item in the survey that teachers have evaluated higher than physical design studio (Fig. 3, left). The final design outcome is considered by the majority to be the same or better than PDS (teachers: 68.4%, students: 78.4%).

It must be noted that not all students had equitable experiences and their opinions might have been affected by their access to the resources required for VDS. Accordingly, the study controlled for external variables, such as internet speed, access to CAD software, and access to adequate computer hardware as evaluated on the 5-point Likert scale by the students. This step was taken to explore the potential effect of such intervening factors, especially for those who reported having negative experiences. Accordingly, a set of Spearman rank correlations were conducted to explore the association between the collected non-parametric ordinal variables (Glasser & Winter, 1961). The results show a slight positive (significant) association between the available computer-related resources and the overall experience; the correlation is relatively lower for the evaluation of the final design product regarding access to CAD software and internet quality. Thus, some of the negative experiences of VDS might have been influenced by these variables (Table 1).

VDS is intrinsically different from PDS in terms of communication medium. Virtual communication poses emerging problems for both students and teachers. The two-sided communication happens in the physical studio over a table or a board; even a very simple working model is a precise and interactive medium and can convey ideas and comments quickly and intuitively. Transforming these interactive communication mediums into VDS was a challenging endeavour. Accordingly, the survey aimed to evaluate students’ abilities to express their ideas in VDS (Fig. 4: right). The responses, here, were different from students’ perspectives. Students found VDS to be similar to PDS (mean = 3.09), whereas teachers reported it to be inferior to PDS (mean = 2.56). A similar trend was observed for working with a digital model instead of a physical model for expressing design ideas (Fig. 4: left; students, mean = 3.40; teachers, mean = 3.06).

The survey asked for the evaluation of tools that students used for drawing, drafting, and visual communication in VDS during the semester. The most common tools were evaluated from ‘1. Not effective at all’ to ‘5. Extremely effective’. The scanning of hand drawings and photography of physical models were also included as an item because it was a common practice among students and lecturers. The mean values are shown in Table 2. It seems that the tools with simple intuitive interfaces (SketchUp) and those known by the majority (AutoCAD and Photoshop) are most effective according to the target group. Coefficient of Variation (CV) is used here to explore the relative dispersion of responses between groups with different sample sizes (Shechtman, 2013). In the case of more complicated tools like Revit, the high CV in responses indicates that those who know the program very well, find it extremely effective whereas others who could not use it effectively found it ineffective. On the contrary, AutoCAD and Photoshop, which are being taught as a part of the curriculum and are the basic go-to applications, show the lowest CV in both groups. Nevertheless, it is difficult to draw a conclusive argument regarding ‘the effectiveness of digital presentation tools in the studio’ due to the high dispersion in the data sets demonstrated by CV (see Shechtman, 2001). However, this dispersion indicates the diversity of preferences for digital presentation tools in the architectural studio.

As suggested by the existing literature, peer learning is one of the fundamental pedagogical attributes of the architectural studio. The survey addressed this issue in two items: informal peer learning (background learning) and the student’s ability to conduct successful group work in VDS compared to PDS. The evaluation of background learning, as reported by the students, was ‘same as PDS’ (mean = 3.06), and teachers evaluated it as inferior to PDS (mean = 2.56). In the open-ended inquiry attached to this question, some students indicated that they learned more from the discussions of their friends’ projects than what they used to learn in PDS (Fig. 5). Two general explanations were given by the students in this regard. First, they could keep working on their projects while listening to the ongoing critique session. Second, the availability of the recording for further revisiting made it possible to pay attention more when the discussion was going on. The class recording also made it possible to revisit the critique session, which many students reported doing. Similarly, students found working on group assignments in VDS to be similar to doing so in PDS (mean = 2.92), whereas teachers evaluated it as worse than in PDS (mean = 2.29). It must be noted that among all measured variables, group work in VDS was ranked lowest by the students when they compared it with group work in PDS.

Based on the initial analysis, the study further explored potential associations between variables affecting the studio (see Fig. 3: experience and outcomes) and the variables that were evaluated by one or both groups as influential (see Figs. 4 and 5: working with 3D digital tools, effective communication, peer learning, and group works). Table 3 shows the side-by-side association (Spearman) between the aforementioned variables for both groups. The results show differences not only between teachers and students but also between the experience and outcome of the studio. The most influential variable associated with the design outcome for both students and teachers is ‘effective communication’. For teachers, maintaining peer connection and group work seems to be the only statistically significant correlation regarding the studio experience, whereas, for students, it remains ‘effective communication’.

**Table 3:** Two sets of Spearman rank correlation coefficients, exploring associations between experience/outcome of design studio and most effective observed variables

Spearman ρ	**Experience:** Overall, how do you evaluate your experience in the virtual design studio during the past semester?			**Outcomes:** How do you evaluate your [your students'] final design products compared with physical design studio?		
	ρ	Sig (2-tailed)	N	ρ	Sig (2-tailed)	N
Teachers						
Working with 3D digital tools	0.019	0.941	18	0.637**	0.004	18
Effective communication	0.408	0.093	18	0.859**	0	18
Peer learning	0.533*	0.023	18	0.774**	0	18
Group works	0.522*	0.032	17	0.727**	0.001	17
Students						
Working with 3D digital tools	0.472**	0	183	0.438**	0	180
Effective communication	0.575**	0	184	0.656**	0	181
Peer learning	0.478**	0	182	0.429**	0	179
Group works	0.503**	0	184	0.490**	0	181

**Correlation is significant at the 0.01 level (2-tailed)

*Correlation is significant at the 0.05 level (2-tailed)

The study also explored the effectiveness of virtual juries as a learning tool in comparison with the PDS juries. Design studio juries are not merely assessment tools, they are an extension of the dialectical feedback on the project and carry a pedagogical role (El-Latif et al., 2020). The ability to present a project to peers and external visiting jurors is a part of architectural professional practice that is learned within the framework of jury evaluation (Lewis, 2013). Virtual space, in this regard, offers a different experience when compared to PDS. In PDS, all projects are on display; furthermore, from students’ points of view, all their documents are seen at once. Whereas in the virtual space, the focus is often on one item of a singular project (Iranmanesh & Onur, 2021). In this case, the virtual jury seems to empower students to focus on the strengths of their project by giving them more control over what is being presented on the screen. The collected data suggests a similar trend to the aforementioned criteria; students seem to evaluate the online jury higher than the teachers do. The gap shows itself clearly, where 40.8% of students evaluate the virtual jury to be better than the physical jury compared to only 5.6% of teachers evaluating it that way (Fig. 6).

It must be noted that this gap might have been influenced by other variables. For instance, the cost of preparing for the physical jury was higher for the students in this case compared to cost of preparation for the online presentation. A significant but weak association was identified between the cost of preparations for the jury and students’ evaluations of the virtual jury (ρ = 0.33). The pedagogical effectiveness for architectural juries has been criticised in the literature (Anthony, 1987; Ward, 1990), but it remains the mainstream practice for the evaluation of design studio outcomes, to date. Perhaps the appeal of the virtual jury to the student reflects a need for the jury to become a more student-centred event.

Despite the positive attitudes toward VDS, both students and teachers expressed a high level of affinity for face-to-face critique sessions and PDS (Fig. 7). More than 77% of teachers and 69% of students reported that they prefer a face-to-face critique session. When asked if they would prefer to have a combination of both methods in the future, the majority agreed that a hybrid method would realize the best of both worlds—creating a studio experience that would supersede either one alone. A similar finding was reported by Rodriguez et al. (2018).

## Conclusion

The current study explored the self-evaluation of the design studio experience and its outcomes from the perspectives of students and teachers. Moreover, various aspects of virtual design studio were analysed, including peer learning, group works, effective communication methods, the integration of the digital tools into the studio workflow, and jury evaluation. The results show that the most critical items associated with both experiences and outcomes of the studio are communications between teacher(s) and student(s) and maintaining peer connections. The communication skills blend into the discussion of students’ fluency with the digital tools that were used for the studio environment.

Identifying the significant differences between students' and teachers' attitudes toward the unplanned VDS is one of the key findings of this study. In general, students evaluated most dimensions of VDS to be similar or better than PDS, whereas the teachers found VDS to be inferior to PDS for most variables. These findings indicate the existence of a generation gap that is embodied in the advent of digitalized communication in the lives of younger generations. The Generation Z students of the faculty have grown up with the internet and have developed the ability to conduct independent self-learning processes. Thus, these students seem to be more flexible in adapting to VDS, whereas the teachers might not find these emerging approaches effective or suitable for what they know as conventional design studio.

The lack of a robust framework might have created some issues for the students and the faculty since the transition to VDS was a sudden change with no prior plan in mind. Nevertheless, the design studio courses moved on, and students accepted and adapted to the conditions of VDS faster than their instructors did. There are lessons to be learned from this experience. The role of the teacher in this process, as suggested by Rancière (1991), could be to motivate students and provide a platform for exploration and self-learning processes. Of course, architecture, as a multi-disciplinary major, requires technical tutoring, but as the results suggest, many of those processes can be learned by providing the right resources and just showing the way. The design studio plays an important role in this regard since it is the nexus of all learning outcomes in the architecture curriculum.

The results of this study indicate a strong association between successful communication and the outcomes of the design studio. The face-to-face critique sessions allow for more casual interactions. An inherent quality of physical space is that it can create dynamic and interactive opportunities (see Fig. 2). However, for the most part, virtual space is limited to bi-directional communication that renders other participants mere observers. The findings indicate that both students and teachers associated successful communication with the successful outcomes of the virtual studio.

Moreover, peer learning is one of the core concepts of architectural pedagogy, and it might come into question regarding VDS. Our findings suggest that students who could maintain their peer communications during distance education were more successful with their design projects. This brings to light the critical importance of digital communication in virtual space. Mere access to good hardware and/or software is shown to be a significant contributor to the experience and outcomes of the studio. Furthermore, the possibility of the teacher’s interaction with the project seems to be a significant variable. In this case, universally known software like AutoCAD and Photoshop, and free, intuitive tools like SketchUp that can be learned quickly were the most successful tools in conducting VDS. The utilisation of these tools might make student–teacher communications easier by narrowing the skill gap between the learners and teachers. Also, it must be noted that VDS might be disadvantageous for some students who are naturally good at sketching and hand drawing.

The design studio evaluation process via jury also requires more attention considering information that arose during the pandemic. The traditional jury often struggled with negativity and stress. The power hierarchy of the jury can potentially make it a stage for teachers’ opinions when it is inherently supposed to be a student-centred event. The preference of students for the online jury highlights some of these issues. Further studies are required to explore how the lessons learned from VDS can elevate the jury evaluation for the betterment of architectural studio pedagogy.

The results of the final items in the survey indicate students’ affinity for the face-to-face critique sessions even though students evaluated their experiences of VDS as similar to or better than PDS. For the most part, students and teachers agree that VDS offers some qualities that are superior to PDS and can highly complement the traditional studio system. This shows the necessity of re-evaluating the models and theoretical frameworks around the design studio pedagogy. As the findings of this study indicate, design studio is a dynamic and resilient entity that survived—and thrived in some aspects—even when its very spatial core was disturbed, As such, the transformation of its traditional approaches does not seem to neglect its core values. It is essential for the pedagogical models of design studio learning to evolve with context, time, innovation, and technology.

A successful educational process in design does not intend to teach every single step leading to an outcome; rather, it is an enabler of discovery and exploration of a student’s capacity. This is what Rancière (1991), Robinson and Aronica (2016), and Sugata et al. (2005) explored for educating the new generation driven by students’ own experiences. Selecting personal approaches and becoming self-aware can affect students’ academic achievements in a very positive way. In the context of design studios, it is necessary to ask questions that will make students curious and have mentors direct them when necessary.

It must be noted that this study was limited by the sample size and special circumstances of the case study. The results that were presented here are promising. Nevertheless, different contextual characteristics, resources, and pedagogical approaches might render different outcomes. In the end, the possibility of using the potentials of virtual space in the future of architectural design studio pedagogy must be acknowledged. Further studies are required to explore the possibility of blended methods for post-COVID architectural education. In this case, the virtual studio was a positive experience for some students and an obstacle for others. This might be the case for the physical studio as well. Thus, redesigning the workflow of architectural design studio in the post-pandemic world might create a more equitable and resilient environment that benefits all students and can cope better with similar future events. Future studies might consider aiming for creating multi-variate statistical models that can address the intrinsic complexities of VDS from different perspectives, such as gender, detailed age ranges, cultural backgrounds, or personality traits.

## Data Availability

The datasets generated during and/or analysed during the current study are available from the corresponding author upon reasonable request.

## Abbreviations

VDS: Virtual Design Studio
PDS: Physical Design Studio
3D: 3 Dimensional
